# A Culturally Targeted eLearning Module on Organ Donation (Promotoras de Donación): Design and Development

**DOI:** 10.2196/15793

**Published:** 2020-01-13

**Authors:** Gerard P Alolod, Heather Gardiner, Chidera Agu, Jennie L Turner, Patrick J Kelly, Laura A Siminoff, Elisa J Gordon, Robert Norden, Theresa A Daly, Amanda Benitez, Ilda Hernandez, Nancy Guinansaca, Lori Ramos Winther, Caroline D Bergeron, Antonette Montalvo, Tony Gonzalez

**Affiliations:** 1 College of Public Health Temple University Philadelphia, PA United States; 2 Department of Social and Behavioral Sciences College of Public Health Temple University Philadelphia, PA United States; 3 Division of Transplantation, Department of Surgery Feinberg School of Medicine Northwestern University Chicago, IL United States; 4 Gift of Life Institute Philadelphia, PA United States; 5 Enlace Chicago Chicago, IL United States; 6 Centro San Bonifacio Chicago, IL United States; 7 Bexar County Community Health Collaborative San Antonio, TX United States; 8 Esperanza Health Center Philadelphia, PA United States

**Keywords:** Hispanic Americans, organ donation, program development, program evaluation, education

## Abstract

**Background:**

As an overrepresented population on the transplant waitlist, stagnated rates of organ donation registration among Latinxs must be redressed. Promotoras (community health workers), who are effective at advocating and spearheading health promotion efforts in the Latinx community, show promise in their ability to educate about organ donation and donor registration.

**Objective:**

This study aimed (1) to develop an interactive, evidence-based program to educate promotoras about organ donation, the need for organ donors in the Latinx American community, and ways to register as deceased organ donors and (2) to train promotoras to lead discussions about organ donation and to promote the act of donor registration.

**Methods:**

In partnership with 4 promotoras organizations, the culturally targeted *Promotoras de Donación* eLearning module was developed based on input from 12 focus groups conducted with Latina women (n=61) and promotoras (n=37). Formative work, existing literature, the Vested Interest Theory, and the Organ Donation Model guided curriculum development. In partnership with the Gift of Life Institute and regional promotoras, the curriculum was designed, filmed, and developed in a visually appealing module interface. The module was beta-tested with promotoras before launch.

**Results:**

*Promotoras de Donación*, available in Spanish with English subtitling, lasts just over an hour. The module comprised 6 sections including various activities and videos, with the curriculum divided into a skills-based communication component and a didactic educational component. Pre- and posttests assessed the module’s direct effects on promotoras’ organ donation knowledge and attitudes as well as confidence promoting the act of donor registration.

**Conclusions:**

This novel, theoretically and empirically based intervention leveraged the existing network of promotoras to promote the act of donor registration. Future research should assess whether the module helps increase rates of donor registration within Latinx communities and reduce disparities in access to transplantation.

**Trial Registration:**

ClinicalTrials.gov NCT04007419; https://www.clinicaltrials.gov/ct2/show/NCT04007419

## Introduction

### Background

Although the number of deceased organ donors in the United States has increased modestly since 2010 [1], a shortage of transplantable organs still remains. The dearth of available organs for transplantation is particularly acute among Latinx populations, an increasingly accepted and common term to represent Hispanic and Latino communities [2]. As of September 2019, over 23,000 Latinxs were candidates on the national transplant waitlist [3], of whom 88% were in need of a kidney transplant [4] because of higher rates of diabetes, chronic kidney disease, and end-stage renal disease compared with non-Hispanic whites. The need among older Latinxs is particularly acute. A large longitudinal study (n=453,162) of kidney transplant candidates showed that the proportion of Latinxs aged 45 years and older on the waitlist grew consistently from 56.4% during the 1995 to 1999 period to 69.8% in the 2010 to 2014 period [5]. Despite the need, only 1508 (14%) deceased organ donors were Latinxs in 2018 [6], although interest in and willingness to donate have been expressed within Latinx populations, including uninsured undocumented Latinx immigrants [7,8].

Since the 2007 revision of the Uniform Anatomical Gift Act making the act of registering as an posthumous organ donor legally binding [9], much like an advance directive or living will, it has become an even more critical first step to increasing the number of individuals converted to actual donors and the number of organs available for transplant. Currently, approximately half of the US population have designated themselves as posthumous organ donors through drivers’ licenses, donor cards, or Web-based registries—an act referred to as donor designation [10]. Donor designation rates among Latinxs have remained much lower than the national average. For instance, a retrospective cohort study of deceased donation decisions found that 6.4% of Latinx participants were designated organ donors [11], and a larger study (n=2070) found that less than 40% of Latinxs had documented their intentions to donate posthumously [12]. Although Latinxs in the United States come from various countries of origin and have distinct immigration histories, the extant literature describes this population as a homogeneous unit. Commonly cited barriers to donation among this population include low levels of acculturation, religious beliefs, mistrust of the medical and organ distribution systems, and cultural taboos regarding discussions about death [13-15]. Encouraging donor designation among all ethnic groups is imperative to improve access to transplantation for all patients on the waitlist. However, increasing the number of Latinx donors is of particular import, given the improved graft outcomes associated with receipt of organs from this subgroup of the population [16].

Prior research has underscored the importance of culturally targeted approaches to addressing the specific health beliefs and transplantation-related needs among Latinx communities. For example, Hu et al’s family-based intervention on diabetes self-management among Latinxs yielded successful outcomes, such as improved blood pressure, higher knowledge about diabetes, healthier behaviors, and lowered body mass index [17]. Gordon et al created and evaluated a culturally targeted website about living kidney donation and transplantation for Latinx patients; exposure to the website in combination with transplant education generated significantly higher transplant knowledge compared with transplant education alone [18]. Other research aimed at increasing organ donation among Latinx populations has suggested women as ideal champions for promoting donor designation, given their dominant position in health-related decision making and their increased willingness to donate and discuss donation with family members [14,19,20].

### Promotores de Salud

Promotores de Salud are a network of Latinx lay health educators (or community health workers) who disseminate health information to their communities through interpersonal channels. Indeed, platicas, or small group discussions typically held in homes or local community centers about different health topics, are organized and guided by promotores. In the 1960s, Promotores de Salud emerged out of necessity in low-income neighborhoods with sizable Latinx immigrant populations, to overcome significant barriers to accessing and utilizing health care services [21]. Promotores are a cost-efficient and effective workforce that expands the reach of the health care delivery system. As such, promotores have typically become the first point of contact between Latinx community members and formal health institutions. Given their knowledge of and membership in local and health care communities, promotores serve as cultural intermediaries, social/emotional supports, peer advocates, connections to available services, and catalysts for community action [22,23].

Promotores de Salud have had demonstrable impacts on health knowledge and behaviors. For instance, they have increased Latinxs’ knowledge of and participation in preventive behaviors for diabetes [24], cervical cancer [25,26], obesity [27,28], and cardiovascular disease [29]. Furthermore, a growing body of evidence suggests promotores can be effectively engaged in the research process and, once trained, can be successful partners in study implementation [30-35]. Promotores have been trained to provide culturally and linguistically appropriate information to Latinx Americans about a wide variety of health-related topics and to promote behaviors to improve health and prevent illness, disease, and disability [36,37]. As more than 80% of these community health workers are female [36] and older Latinxs are increasingly overrepresented on the national waitlist [5], we believed that this existing network of promotores could be leveraged to educate mature Latinas (aged 50 years and older) in their respective communities about organ donation. We also anticipated that mature Latinas would be influenced to designate themselves as posthumous organ donors and support donation within their families, as women are the primary source of information about health and health care in Latinx families. In addition, women, in particular, express greater willingness to register as deceased organ donors [14,19] and are more likely to register, be converted to actual donors, and discuss donation with their families [14,19,20].

### Web-Based eLearning Modules

Web-based electronic learning (eLearning) platforms (ie, learning management systems) are highly variable in their features and methods of instruction [38]. Such customizability allows developers to remain sensitive to the practical and cultural needs of the target audience and helps ensure ease of use and engagement with material. Interactive computer- and Web-based public health and patient education trainings [38,39] have successfully addressed the learning needs of older adults [40-42], underserved populations [18,43-48], and those with chronic communicable disease [49], especially when culturally informed [50]. Previous studies employing Web-based interventions with targeted features to effectively train *promotoras* have demonstrated that eLearning leads to wider reach [51] and impact in Latinx communities [52]. The Office of Minority Health (OMH) of the US Department of Health and Human Services’ creation and promotion of a Web-based training for *promotoras* about best practices for promoting health in their communities underscores this fact [53].

### Promotoras de Donación

We developed the *Promotoras de Donación* Web-based eLearning module to increase *promotoras’* knowledge of organ donation and transplantation and to improve their communication skills and confidence discussing and promoting organ donation with mature Latinas. The feminine form of the term *promotor* was chosen because most community health workers are female, as are our target audiences. The module is designed to increase rates of donor designation within Latinx communities across the United States and to ultimately reduce disparities in transplantation among Latinxs. This paper describes our development of the *Promotoras de Donación* eLearning module, including the formative work involved in the module’s development and the module’s theoretical underpinnings. We present the module design, structure, cultural foundations, the results of beta-testing, and the plan for modifying the module in response to the feedback received. The paper concludes with challenges to the development of the module; limitations; and plans to evaluate the module’s direct, indirect, and sustained effects.

## Methods

### eLearning Module Development

#### Study Team

The research team comprised Temple University faculty and staff including the principal investigator with expertise in interpersonal health communication and organ donation and transplantation (HG), 2 coinvestigators—a medical anthropologist with expertise in developing culturally targeted interventions in organ transplantation and donation from Northwestern University (EJG) and an internationally recognized expert in deceased organ donation (LS)—a creative coordinator (GPA), a project coordinator (CA and JT), and an undergraduate research assistant (PJK). The Gift of Life Institute, a Philadelphia-based international training center with extensive experience designing and developing Web-based educational programs for donation professionals, aided in curriculum design and module interface development (TD and RN). Textbox 1 illustrates the steps in the module’s development.

**Steps in module development, implementation, and evaluation.**

**Formative research (September 2016–August 2017)**

1. Recruited 4 promotore organizations as community-based partners.

2. Conducted cognitive interviews to develop and refine focus group moderator’s guides.

3. Conducted and analyzed 12 focus group discussions—8 with mature Latinas and 4 with promotoras (N=98).

**Module development (September 2017–August 2018)**

4. Theoretically grounded in Organ Donation Model and Vested Interest Theory.

5. Curriculum development—integrated theories, extant literature, and formative research findings.

6. Script writing—identified didactic and skills-based sections of the module and strategically placed embedded media.

7. Design features developed in consultation with Gift of Life Institute and ensured cultural and linguistic targeting

8. Filming preparation including hiring and training actors and coordinating with production team.

9. Filming, editing, and embedding content in learning management system.

**Implementation (September 2018–December 2018)**

10. Soft launch for testing with Temple University team.

11. Beta-test (N=10) for acceptability, cultural appropriateness, platform navigability.

12. Integration of beta-test results into final version of module.

13. Assessment of direct effects of module on Promotoras’ (N=40) organ donation attitudes and knowledge, donor registration behaviors, and confidence discussing donation.

14. Assessment of indirect effects of module on mature Latinas’ organ donation knowledge and attitudes, and donor registration behaviors.

15. Assessment of sustained effects of module on Promotoras’ organ donation attitudes and knowledge, donation behaviors, and confidence discussing donation

We partnered with 4 *promotoras* organizations representing 3 geographically diverse locales and heterogeneous Latinx populations: the Bexar County Community Health Collaborative (San Antonio, Texas), Enlace Chicago and Centro San Bonifacio (Chicago, Illinois), and Esperanza Health Center (Philadelphia, Pennsylvania). Each site was selected based on established relationships with local *promotoras* organizations, cost, and representation of Latinxs from different countries of origin. Leadership and *promotoras* from each organization met virtually with the Philadelphia-based research team on a quarterly basis for updates on study progress and discussion of challenges and next steps. Partners also provided feedback on study processes including recruitment plans and materials, the content of study instruments, Spanish translations, branding, module content, and beta-testing. *Promotoras* organizations guided the peripheral, evidential, linguistic, and sociocultural strategies employed to ensure the resulting module was culturally targeted and linguistically congruent [54].

#### Theoretical Foundation

**Figure 1:**
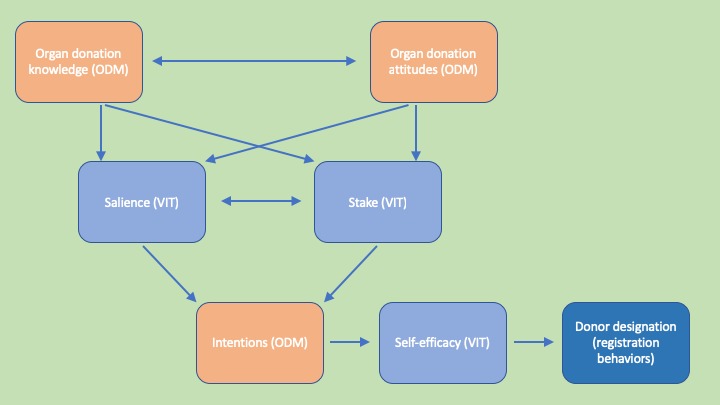
Theoretical framework of *Promotoras de Donación*. Organ Donor Model (ODM) Vested Interest Theory (VIT).

*Promotoras de Donación* is theoretically rooted in the Organ Donation Model (ODM) and Vested Interest Theory (VIT). Figure 1 provides a pictorial representation of the module’s theoretical framework. Proposed by Morgan and Miller [55,56], the ODM is grounded in the Theory of Reasoned Action (TRA) and highlights the importance of donation intentions on the act of becoming a designated organ donor. The model posits that attitudes toward and knowledge about organ donation influence designation intentions [55,56]. The model has found support in empirical tests among non-Hispanic whites and, in a slightly modified form, among African Americans [57-60]. In addition, constructs from both Azjen’s Theory of Planned Behavior, a descendent of the TRA, and VIT have been useful in explaining differences in perceptions about living and nonliving organ donation among Hispanics [61].

VIT similarly delineates the relationships among and between constructs thought to predict donor designation [61]. VIT has been used to help explain the discrepancy between highly positive attitudes toward donation and low rates of donor designation. Specifically, VIT posits that the relationship between donation-related attitudes and low donor designation rates is moderated by perceptions of personal importance (ie, vested interest). VIT delineates 5 dimensions of *vested interest*: *stake* (perceived positive and negative consequences associated with a given issue), *salience* (vividness and accessibility of the issue), *self-efficacy* (perceived ability to overcome behavioral barriers), *certainty* (belief that the perceived consequences will be realized), and *immediacy* (belief in the imminent manifestation of those consequences) [62]. Scholars recommend the application of VIT to understand organ donation designation behaviors, focusing on the first 3 dimensions and indicating that self-efficacy in designating oneself as a donor proved a strong predictor of donor registration [63-65]. We conceptualize VIT constructs of stake, salience, and self-efficacy as representing awareness of the benefits of donation, recognition of the importance of and need for organ donors in the Latinx community, and confidence in making a decision about donation and donor designation.

These theoretical constructs are incorporated throughout the module. Short videos created by the Health Services and Resources Administration’s Division of Transplantation as well as narratives (ie, anecdotal and expert testimony) and statistical messages (ie, data about the need for organ donors) are included to increase and enhance *promotoras’* knowledge and attitudes toward organ donation and donor designation. To increase self-efficacy, the module demonstrates the skills needed to introduce and manage *platicas* about organ donation through videos that model such discussions. VIT constructs are included in illustrations of the discrete skills needed to effectively present the need for Latinx donors (salience), articulation of the benefits of donation (stake), and presentation of instructions for donor registration (self-efficacy).

#### Module Structure and Curriculum

**Figure 2:**
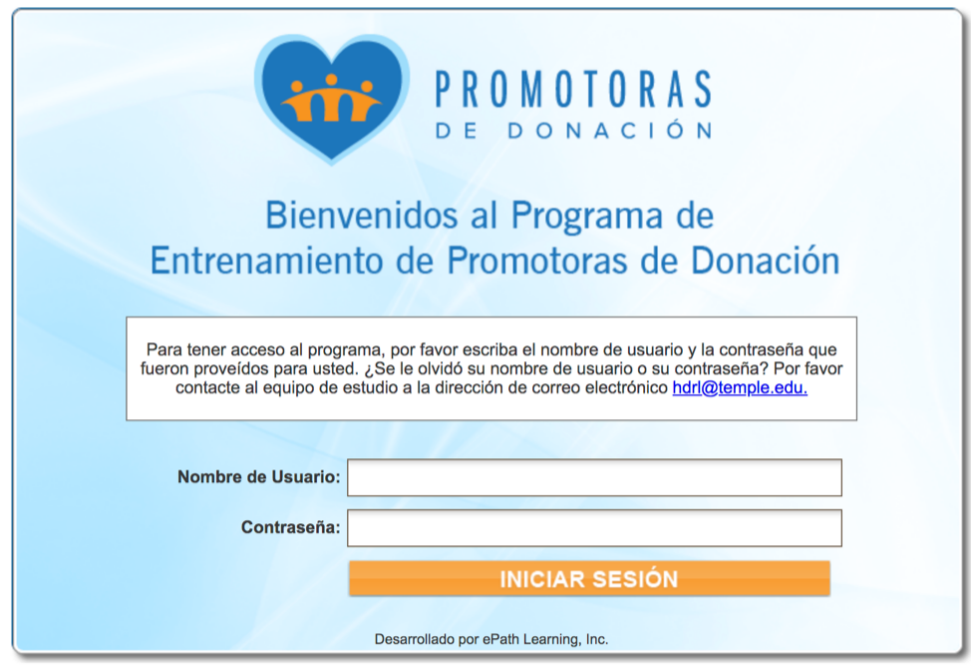
Screenshot of the login page of *Promotoras de Donación* to illustrate the branding standards.

*Promotoras de Donación* was designed to be a highly engaging and interactive online learning experience (see Figure 2). Interactivity is an essential feature of learning in online environments and can support the pace at which the content is presented and enacted, development of associations between existing knowledge and acquisition of new information, reinforcement and refinement of newly acquired skills, guidance through the content learned, and enjoyment of the educational experience [66-68]. In addition, we accommodated multiple learning styles by incorporating a variety of pedagogic approaches into the module. For instance, written information and narration is accompanied by a public educational video promoting organ donation by depicting a canine pet following the deceased owner’s transplanted heart to another person [69]; a video describing the organ donation process [70]; an animated video of how to register as an organ donor; a true/false activity; and video testimonials from a donor mother, a liver transplant recipient, and a transplant expert. See Multimedia Appendix 1 for snapshots of its general elements.

Produced in Spanish (with English subtitles), the module was designed to achieve 3 specific learning objectives. After completing the module, we anticipated that learners would (1) demonstrate high levels of knowledge of organ donation and donor designation; (2) confidently engage members of their community in conversations about organ donation; and (3) effectively persuade individuals to designate themselves as posthumous organ donors via a donor card, drivers’ license, or online registry. Guided by a female narrator *(narradora)*, the module originally comprised 6 sections (*secciones*): (1) introduction to organ donation and transplantation, (2) how to register as an organ and tissue donor (3) how to talk with others about organ and tissue donation, (4) a true/false activity, (5) how to promote donor registration, and (6) conclusion. Table 1 lists the subjects covered within each section and the embedded media before the final version of the module after beta-testing.

**Figure 3:**
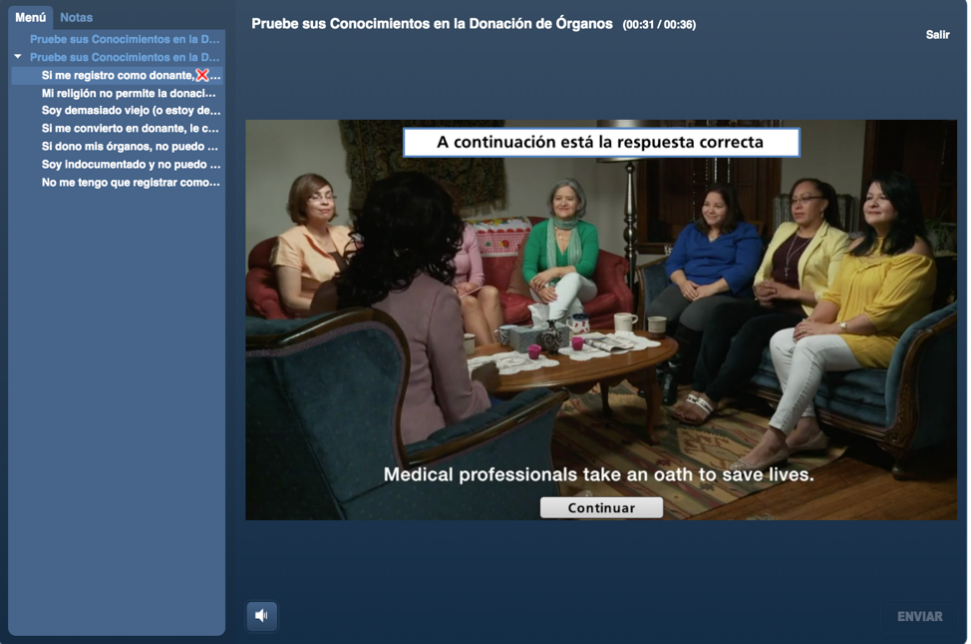
Screenshot of a platica dramatization from the *Promotoras de Donación*.

The curriculum consists of 2 components: a skills-based communication component and a didactic educational component. The communication skills needed to effectively discuss donation and share educational content were modeled in videos depicting a *promotora* leading a *platica* about organ donation and transplantation and promoting donor registration (see Figure 3). We employed and trained 7 *promotoras* from our local partnering organization to portray the roles of the *promotora* and *platica* attendees. In collaboration with the partnering organizations, scripts were developed to train the *promotora* actors and incorporate cultural concerns revealed in the formative focus group discussions throughout the module. The actors were trained weekly in October and November 2017, and the videos were filmed in December 2017. The videos and activities were edited, rendered, and embedded within a learning management system hosted by the Gift of Life Institute.

The skills-based component provides instruction on the communication skills needed to effectively engage participants during *platicas* about organ donation. To be effective communicators, *promotoras* must acquire the skills needed to discuss organ donation and promote donor designation and be motivated to use the skills [71,72]. The *platicas* depicted in the module are intended to model these skills and build communication self-efficacy or confidence in starting the discussion about donation, addressing concerns raised by attendees, and promoting the act of donor designation using persuasive but noncoercive language. In addition, the program trains *promotoras* to exercise communicative tasks such as broaching the topic of organ donation, providing basic information, highlighting the benefits of donation to society, emphasizing the need for Latinx donors, promoting and addressing concerns about donor designation, providing instruction on how to register as an organ donor, answering questions, and closing the discussion.

**Table 1:** *Promotoras de Donación* module content.

Section and content		Embedded media
**Section 1: Introduction to Organ Donation and Transplantation**		
	Review of learning outcomes	Argentinian PSA^a^ depicting an older man and his dog
	Benefits of donation to donor, recipient, donor family, society	HRSA^b^ Video “Organ Donation and Transplantation: How Does it Work?”
	Information on the need for donors, the national transplant waiting list, the donation process	—^c^
**Section 2: How to Register as an Organ and Tissue Donor**		
	Information on how to register as an organ donor and the importance of family discussion about donor designation	Testimonial: Latinx organ recipient
**Section 3: How to Talk to Others About Organ and Tissue Donation**		
	How to prepare for a platica	Platica dramatization
	Communication skills: assess existing knowledge, ask open-ended and probing questions, provide factual information, correct myths and misinformation, and invite questions	Testimonial: transplant physician
	Addressing myths: medical negligence, irreversibility of brain death, moral failure of organ recipient, undocumented immigrant eligibility, religious objections, black market organ trafficking	Testimonial: Latina mother of a deceased donor
	How to converse with the family about end of life decisions	—
**Section 4: A True/False activity**		
	Statements concern: medical negligence, prohibitive religious beliefs, prohibitive age and illness status, donation costs, infringed funeral wishes, inelligibility of the undocumented person, precedence of advanced directives	True/False activity
**Section 5: How to Promote Donor Registration**		
	Communication skills: gauging support, emphasize importance, correct myths and misinformation, support individual choice, remain nonjudgmental, provide instruction on how to register	Second part of platica dramatization
	Methods of donor designation: DMV^d^, online, a donor card	—
	Addressing common reasons people do not designate	—
	How to close the platica	—
**Section 6: Conclusion**		
	Reminder to revisit *Promotoras de Donación* as needed	—

^a^PSA: public service announcement.

^b^HRSA: Health Resources and Services Administration.

^c^Not applicable.

^d^DMV: Department of Motor Vehicles.

The didactic component provides basic information about organ donation and transplantation and the need for donors in the Latinx community. It also addresses concerns about organ donation and donor designation commonly shared by the target population, as revealed through the extant literature and a series of 12 focus groups we conducted with *promotoras* (n=37) and older Latinas (n=61) to inform the module’s development [73]. Focus group participants reported myths and misinformation about organ donation, medical mistrust, and family aversion of discussing sensitive topics as some of the major barriers to organ donation and donor designation. Participants also acknowledged the need for transplantable organs and increased education about the topic within Latinx communities.

The didactic component featured several topics during the dramatized *platicas*. *Platica* attendees asked about stories they heard about the black market, beliefs about health care providers doing less to save the lives of designated organ donors, worries about the incompatibility of organ donation and religious beliefs, the impact of being undocumented immigrants on the ability to receive a transplant, and how to handle discussions about organ donation with family members. The module depicts the *promotora* skillfully and thoughtfully responding to the attendees’ questions and fears about organ donation in Latinx communities and drew on their shared experiences and cultural concerns to deliver information about organ donation and to promote donor designation. The true/false activity was designed to test retention of and reinforce the information provided throughout the module.

A primary goal of module development was ensuring its cultural appropriateness and receptivity by the target population. Throughout the scriptwriting and postproduction process, Spanish-speaking Latinx consultants and our Latinx community partners offered guidance to ensure that the module and its content were realistic, culturally targeted, and linguistically congruent [46]. Our collaborators helped to refine the dialog and educational material by providing insight on focus group data reflecting cultural beliefs and myths about donation. *Promotora* actors represented different Latin American countries and accents to model realistic donation discussions that reflect the variety of Latinx nationalities represented in the United States. The module’s script also incorporated specific statistics about the need for donors in the Latinx community. Table 2 details the specific design and content features that were included to ensure the module’s cultural and linguistic appropriateness [55].

A certificate of completion is provided upon module conclusion. The *Promotoras de Donación* module is accompanied by an Educator’s Manual containing a list of potential discussion topics, commonly asked questions about donation and their answers, and sample language to sensitively correct myths about donation.

**Table 2:** Strategies for achieving cultural and linguistic appropriateness.

Strategy	Design feature	Content feature
Peripheral	Branding and color schema	Use of Latina narrator (narradora)
Evidential	—^a^	Videos and testimonials from Latina organ recipient, donor family, and transplant physician
Linguistic	—	Spanish with English subtitles; available Spanish on screen text
Constituent involving	Feedback from promotoras and leadership from promotora organizations	Integrated feedback from promotoras and leadership from promotora organizations, promotoras as module actors
Sociocultural	—	Module addresses commonly cited myths about organ donation found in literature and focus group data

^a^Not applicable.

### Beta-Testing

#### Procedure

The original version of the module was timed at 1 hour, 14 min, and 29 seconds. The module underwent beta-testing from September 17, 2018, to October 12, 2018. Beta-testing involved full navigation of the module and completion of a postmodule questionnaire measuring user experience and soliciting feedback for improvement. A total of 10 people participated in beta-testing including 3 members of the leadership teams at partnering *promotoras* organizations, 6 *promotoras*, and 1 partnering organization administrator. *Promotoras* participating in beta-testing were compensated with a US $25.00 gift card.

#### Survey

The beta-test questionnaire comprised 40 questions evaluating perceptions of the module; cultural sensitivity; platform navigability; and sociodemographics including job role, length in job role, and preferred language spoken at home. See Multimedia Appendix 2 for the beta-test questionnaire.

Perceptions of the module were assessed through 13 5-point Likert questions (1—*strongly disagree*; 5—*strongly agree*) ascertaining organization, length, realism, and interest in the material covered. Cultural competency was assessed through 5 4-point Likert-type questions (1—*strongly disagree*; 4—*strongly agree*) adapted from the Cultural Sensitivity Assessment Tool [74]. Participants were asked to assess website and module navigability using 5 4-point Likert-type questions (1—*strongly disagree*; 5—*strongly agree*) adapted from the Informational Fit-to-Task Items [75].

Participants were then asked to identify which segments (ie, true/false activity and testimonials) could be shortened or removed through 8 3-point Likert-type questions (1—*definitely remove*; 3—*definitely do not remove*). A single 5-point Likert-type item measured respondents’ rating of the overall quality of the module (1—*poor*; 5—excellent). The questionnaire ended with 5 open-ended questions that asked respondents to indicate suggestions for improving the training program, features that were missing or did not feel right, the most important message for people unfamiliar with the topic of organ donation and transplantation, and the most and least liked aspects.

## Results

### Quantitative Results

Most beta-testers spoke Spanish and English equally at home (7/10) and identified as *promotoras* (6/10); the remaining 4 were leadership from the 4 partnering organizations. Most respondents somewhat or strongly agreed that the information presented was novel (7/10), felt the module activities helped them learn (9/10), enjoyed module activities (9/10), felt ready to teach others about organ donation and transplantation after viewing the module (8/10), would recommend this training to other *promotoras* (9/10), and felt the module was too long (6/10). Most respondents (9/10) agreed or strongly agreed that the module was free from stereotypes about the Latinx community, that the people in their community would believe the messages in the module were from credible sources, and that the messages in the module address commonly shared organ donation and organ transplantation myths in the Latinx community. Regarding website navigability, all respondents disagreed with the statement, “the website was too complex”; all respondents agreed or strongly agreed that the website was easy to use and that they liked the look and feel of the website. Most respondents (9/10) disagreed that the words, phrases, and expressions used in the module were too technical for the average *promotora,* and most respondents (8/10) agreed or strongly agreed that *promotoras* would find the website easy to use.

Half of the respondents (5/10) suggested removing the true/false activity, and 40% (4/10) of the respondents suggested removing the interview with a transplant expert. Most (8/10) respondents recommended definitely not removing the segments describing personal experiences with donation (mother of an organ donor) and transplantation (liver transplant recipient).

### Qualitative Results

In addition to scale items, responses to open-ended questionnaire questions provided rich, contextualizing data suggesting improvements to the original module. Table 3 presents the qualitative questions and a selection of corresponding responses.

We gleaned several lessons from themes emerging from the open-ended responses. First, although beta-testers stated the module was informative and interactive, most found the module to be too lengthy and offered suggestions on content that could be shortened or edited out. Moreover, they noted that the most important messages of the module included life-saving opportunities of organ donation and the need for Latinx donors; they also liked the testimonials included in the module.

**Table 3:** Selected responses to open-ended questions.

Open-ended question	Selected responses
Please use the space below to write down any suggestions on ways to improve the training program.	“This was very well done. I learned a lot and registered as an organ donor right at the beginning after the video with the dog.” “I wouldn't entirely remove the videos with the doctor and the mother who lost her son, but would recommend cutting them down. I'd also cut down some of the conversation led by the promotora. That section was definitely too long.” “The module is very well done, with interesting ways to present the topics and with several personal stories. I did not feel that it was boring. To improve it, I wonder if there is any way to connect the promoters [sic] with a local organization if they want to learn more or become more involved in this area.”
What, if anything, was missing or did not feel right for you in this module?	“I can't think of anything really. There was just the length that was way too long for me.” “I was not sure if the promotora was saying “traNsplante” or “trasplante”. The correct pronunciation would be trasplante for a transplant.”
What do you think is the most important message in this video for people who are unfamiliar with the topic of organ donation and transplantation?	“One person can make a difference.” “That organ donation/transplantation saves lives and is definitely something to consider and talk to others about.” “The video explains the importance of organ donation in the Latino community and the need that exists in the community of more Latino organ donors.” “The myths. The amount of persons a donor can help and tissue, skin, etc. donations”
What did you like most about the module?	“I like that the module is interactive and presents the information in several ways.” “The information that older people [can be] included as donors.” “The real people interviews.” “The participation of the people in the talk and the confidence that the promoter gave.” “Informative in a relaxed, friendly environment”
What did you like least about the module?	“A lot of explanations from the host... some of the content was then repeated in the videos (e.g., about how to register as an organ donor).” “It needs to be cleaned up a bit to be a bit shorter.”

## Discussion

### Principal Findings

*Promotoras de Donación* is the first online training program about organ donation designed specifically for promotoras. We employed a rigorous process to develop this eLearning module. Specifically, following well-established guidelines, we incorporated findings from the extant literature research on organ donation beliefs among Latinx Americans, relied on established theories for guidance, and engaged promotoras and leadership from promotora organizations for advice and feedback throughout the process. As no research had yet examined the knowledge of and attitudes toward organ donation and transplantation or the act of donor designation among either promotoras or mature Latinas—our target audiences—we conducted a series of focus group interviews to elicit this information [74]. The findings of the group interviews were integrated in the design and content of the module. The result was the creation of an empirically and theoretically based, culturally and linguistically appropriate eLearning module designed through a collaborative partnership with promotoras and promotora organizations.

Beta-testing offered valuable feedback about user experience, acceptability, and content. To prepare the module for implementation and evaluation, we made several modifications. For example, we modified the module by increasing the font size for the true/false activity, enabling sensible advancement to subsequent sections for ease of module progression, including removing numbered sections on the menu bar, adding English subtitles, and disabling user manipulation of the progress bar. To reduce module length, we edited testimonials from the mother who had donated her deceased son’s organs and the transplant expert. We moved the animation depicting 3 ways to donate and the true/false activity to appear on the homepage after module completion as supplemental, rather than required material. After revisions, the *Promotoras de Donación* module lasts 1 hour, 3 min, and 38 seconds, with 7 min and 49 seconds of supplemental content. Table 4 provides a comparison of the original and final versions of the module.

**Table 4:** Design elements in original and final versions of the module.

Design element	Original version	Final version
Subtitling	Absent	Added in English with transcript of script available at all times in a togglable tab
Advancement through the module	Unclear and confusing numbering	Logical numbering of each section Explicit instructions to progress through module
Progress bar	User able to manipulate	Disabled ability to manipulate
Older man and the dog video	Section 1	No change
HRSA^a^ video: “Organ Donation and Transplantation: How Does it Work?”	Section 1	No change
Testimonial: Latinx organ recipient	Section 2	No change
Platica dramatization	Sections 3 and 5	Appear closer together in sections three and four
Testimonial: transplant physician	Section 3	Retained in section three but tailored testimonial to address concerns of a black market to reduce length
Testimonial: Latina mother of a deceased donor	Section 3	Retained in section three but reduced in length
True/False activity	Section 4	Removed from module and included as a supplemental activity Font sized increased
Three ways to donate animation	Embedded within the module	Removed from module and included as a supplemental activity

^a^HRSA: Health Resources and Services Administration.

### Next Steps

The module is currently undergoing evaluation of its direct, indirect, and sustained effects. In the first phase of evaluation, we will recruit 40 *promotoras* (10 per partnering site) to complete the *Promotoras de Donación* module and associated pre- and postsurveys. The presurvey will be administered before participants are given access to the module, and the postsurvey will be administered upon module completion; both surveys have been translated into Spanish and will be administered online via Qualtrics. The surveys assess changes in organ donation knowledge and attitudes and confidence communicating about and promoting the act of donor designation.

After completing phase 1, the same *promotoras* will be invited to enroll in the second phase of the evaluation, conducted to test the indirect effects of the module on mature Latinas’ organ donation knowledge, attitudes, and intentions to register as posthumous organ donors. Specifically, trained *promotoras* will lead 2 *platicas* in their respective communities with up to 8 mature Latinas in each session. Before leading the *platicas,* participating *promotoras* will be apprised of the study protocols and trained in human subject’s protections. *Promotoras* will administer anonymous paper-pencil surveys to mature Latinas before beginning the discussion and before attendees leave the *platica*.

To measure the module’s sustained impact on *promotoras’* organ donation knowledge and attitudes as well as communication confidence, participating *promotoras* will complete self-administered surveys before leading their first *platica* and after completing both. If the intervention demonstrates significantly increased rates of donor designation, our Web-based training will be disseminated widely to *promotoras* organizations across the United States and a petition will be made to the HHS OMH to include the module in its existing eLearning program for *Promotores de Salud* [53].

### Challenges

Several challenges arose in the development of *Promotoras de Donación* module. Reliance on multiple sources (ie, focus groups, partner organizations, and beta-testers) for input on module content and format increased module production and revision time frames. Although the feedback was critical to ensure the cultural appropriateness and relevance of the module for the target population, we underestimated the time needed to solicit, review, and incorporate feedback from every collaborating partner. Turnover in staff also contributed to the delays. Our original creative coordinator, who was a fluent Spanish speaker with advanced training in research, resigned necessitating the search for Spanish-speaking consultants in each state to facilitate the focus groups and analyze the resulting data. We also lost 2 study coordinators during critical periods of module development—the first was during finalization of the original version and the second was just before final modifications were made. Other researchers developing similar community-based eLearning interventions would do well to anticipate longer production timetables by adding 2 to 3 months before the launch of the intervention.

The decision to incorporate multiple sources of feedback on the module’s content also led to a longer overall running time than was originally planned. Although the final version of *Promotoras de Donación* was shortened by making original components supplemental, the module still lasts over 63 min in its final form with close to 8 min of supplemental material. This is considerably longer than initial plans of an approximately 20 to 30 min eLearning module. Even though we have yet to see if the increased length of the module will have any demonstrable impact on participant’s experience and satisfaction, the substantial increase in content required additional time for editing, additional hosting space to accommodate the size of media files, and troubleshooting with the Gift of Life Institute’s technological capabilities.

### Conclusions

*Promotoras de Donación* is a novel, culturally targeted intervention firmly grounded in theory, extant literature, and formative research. In response to a lack of knowledge of organ donation and low willingness to register as a posthumous organ donor among Latinxs, the module aims to engender more favorable attitudes toward organ donation, improve knowledge levels, and build confidence discussing donor designation among promotoras as means of increasing registration rates within Latinx communities. The results of our beta-testing demonstrated strong user acceptability of the platform and the content. Furthermore, the module may be dispersed to Latinx communities across the nation at low cost and has considerable potential to increase the number of Latinx registered as deceased organ donors, thus helping to reduce the ongoing national shortage of transplantable organs.

## Appendix

Multimedia Appendix 1 Screenshots of the Promotoras de Donación eLearning module.

Multimedia Appendix 2 Post Module Questionnaire.

## Abbreviations

HRSA/DoT: Health Resources and Services Administration’s Division of Transplantation
ODM: Organ Donation Model
OMH: Office of Minority Health
TRA: Theory of Reasoned Action
VIT: Vested Interest Theory
