# Supplementary material for: A Culturally Targeted eLearning Module on Organ Donation (Promotoras de Donación): Design and Development
Source: J Med Internet Res. 2020 Jan 13;22(1):e15793. doi: 10.2196/15793 (PMC6996759; doi:10.2196/15793)

PROMOTORAS DE DONACIÓN

Multimedia Appendix 1

Screenshots of Key Features

Screenshot 1

The login page

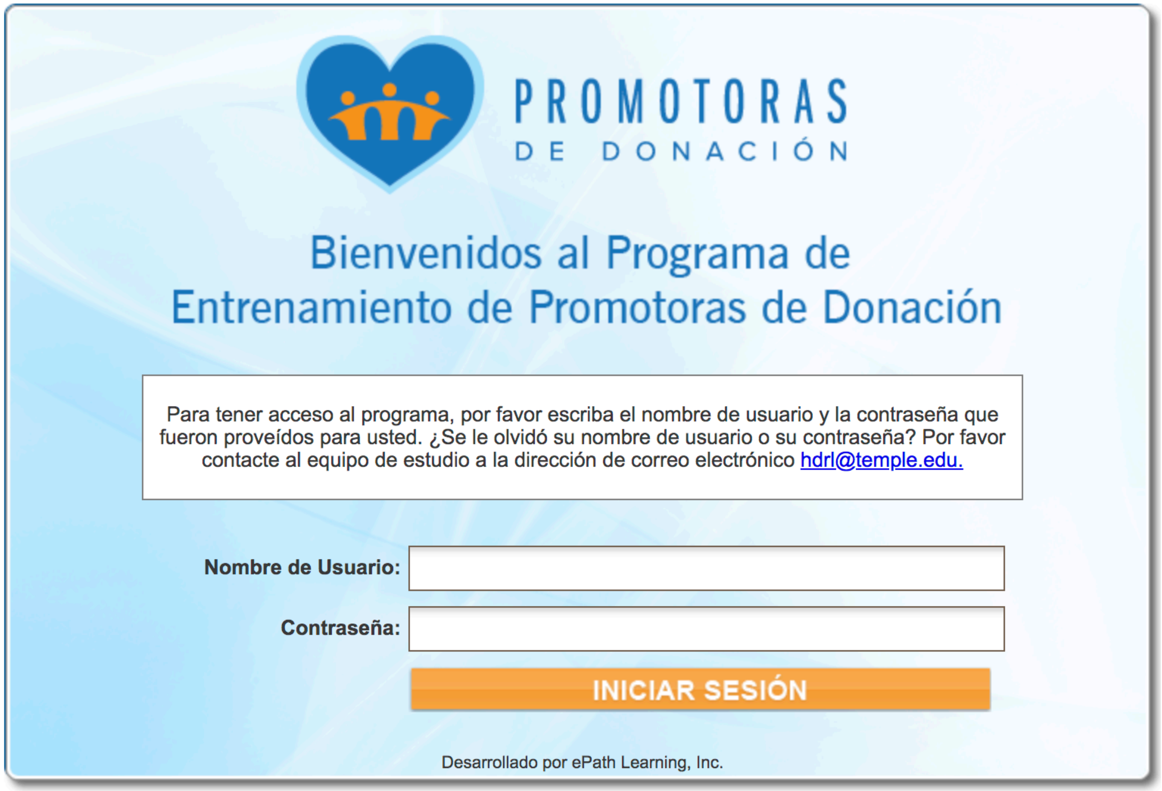

Screenshot 2

The homepage

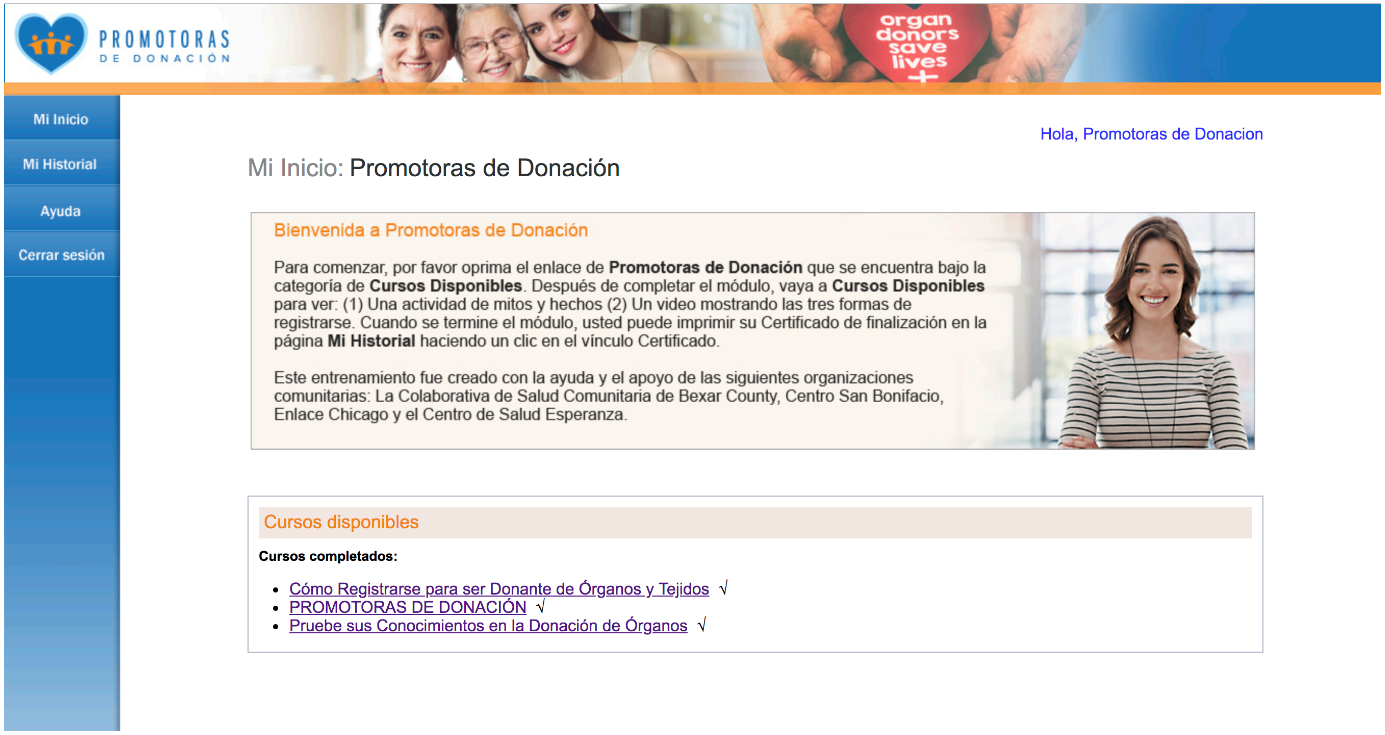

Screenshot 3

The welcome screen

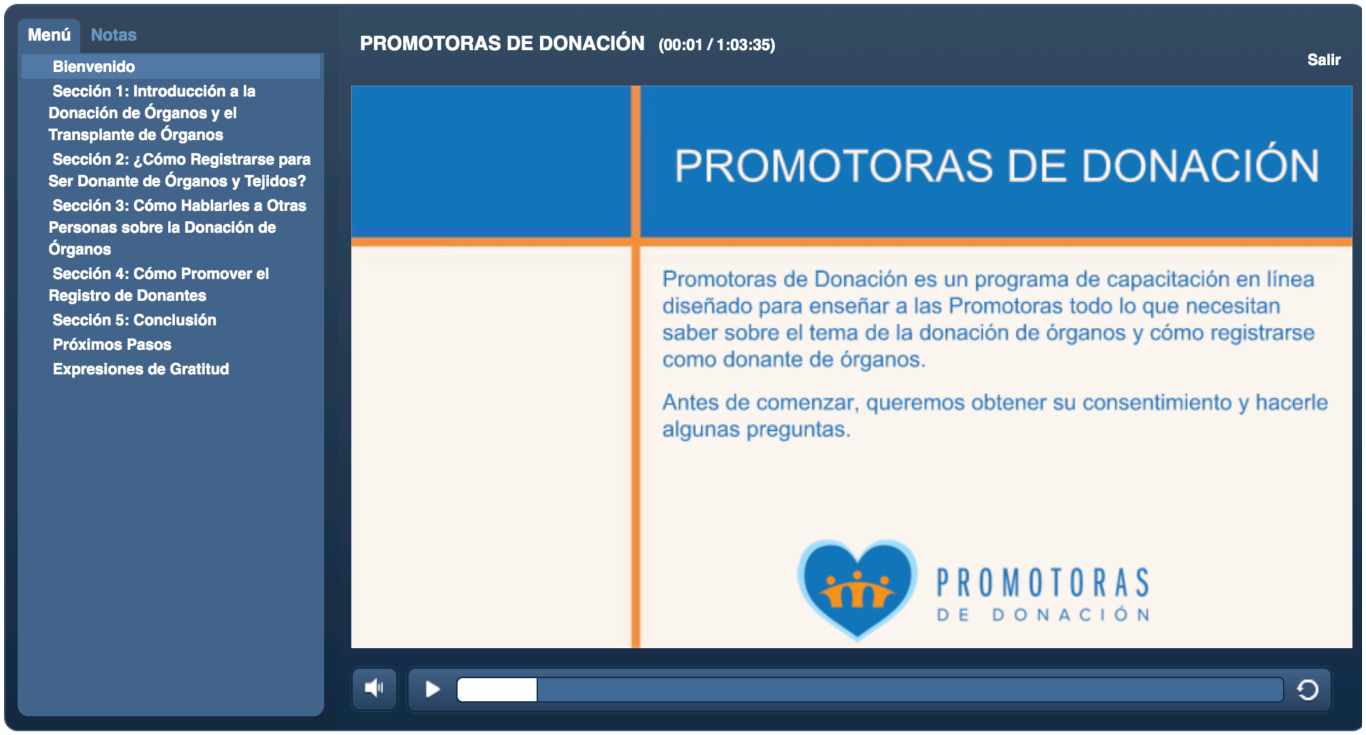

Screenshot 4

The educational video, “The Man and the Dog”, that promotes organ donation

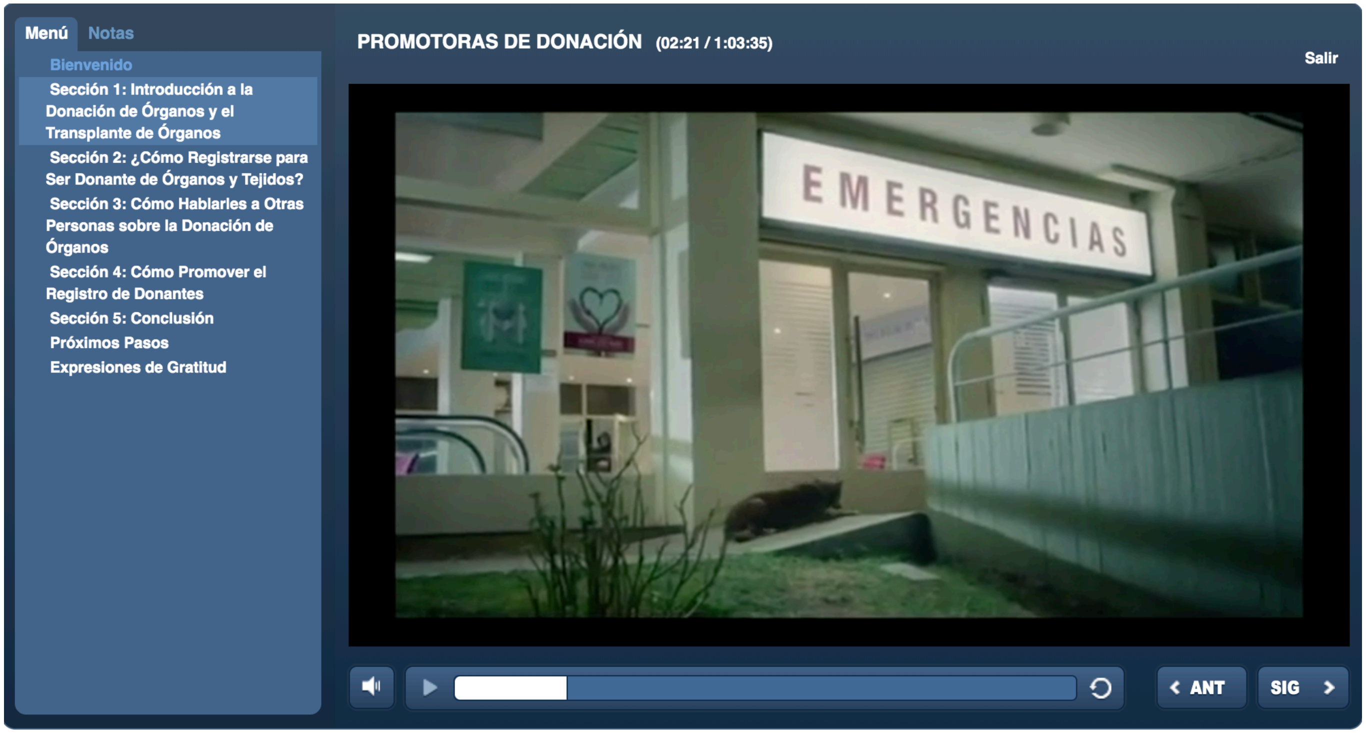

Screenshot 5

The narrator reviewing the learning objectives of *Promotoras de Donación*

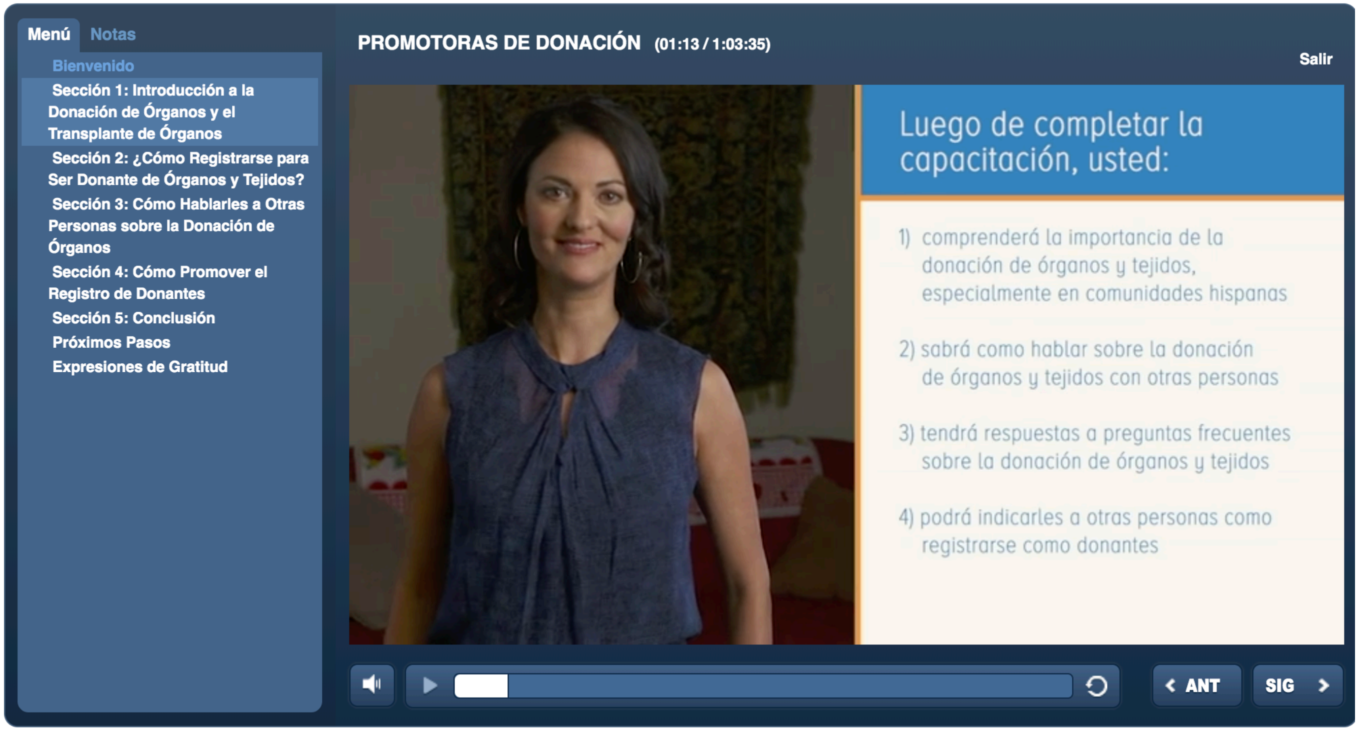

Screenshot 6

The Health Resources and Services Administration video describing the organ donation process

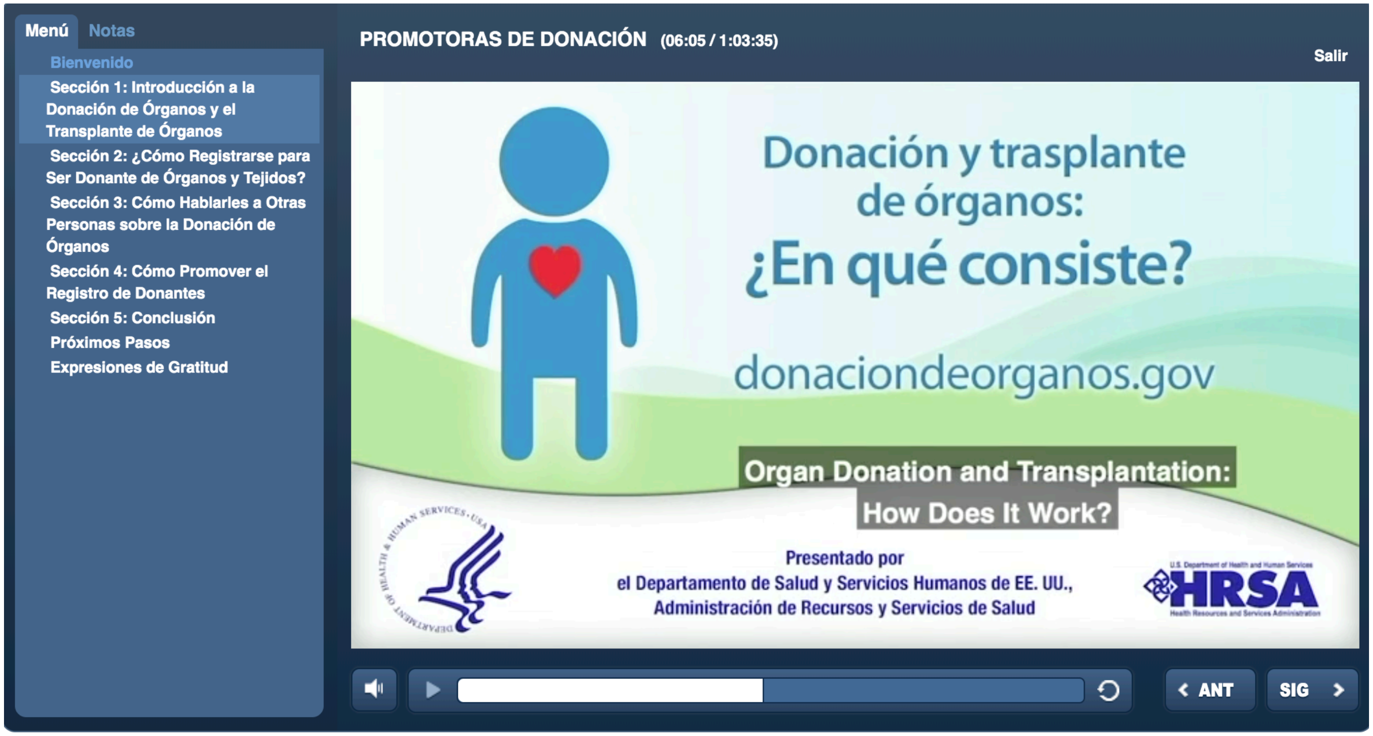

Screenshot 7

The *promotora* opening the *platica* by introducing herself

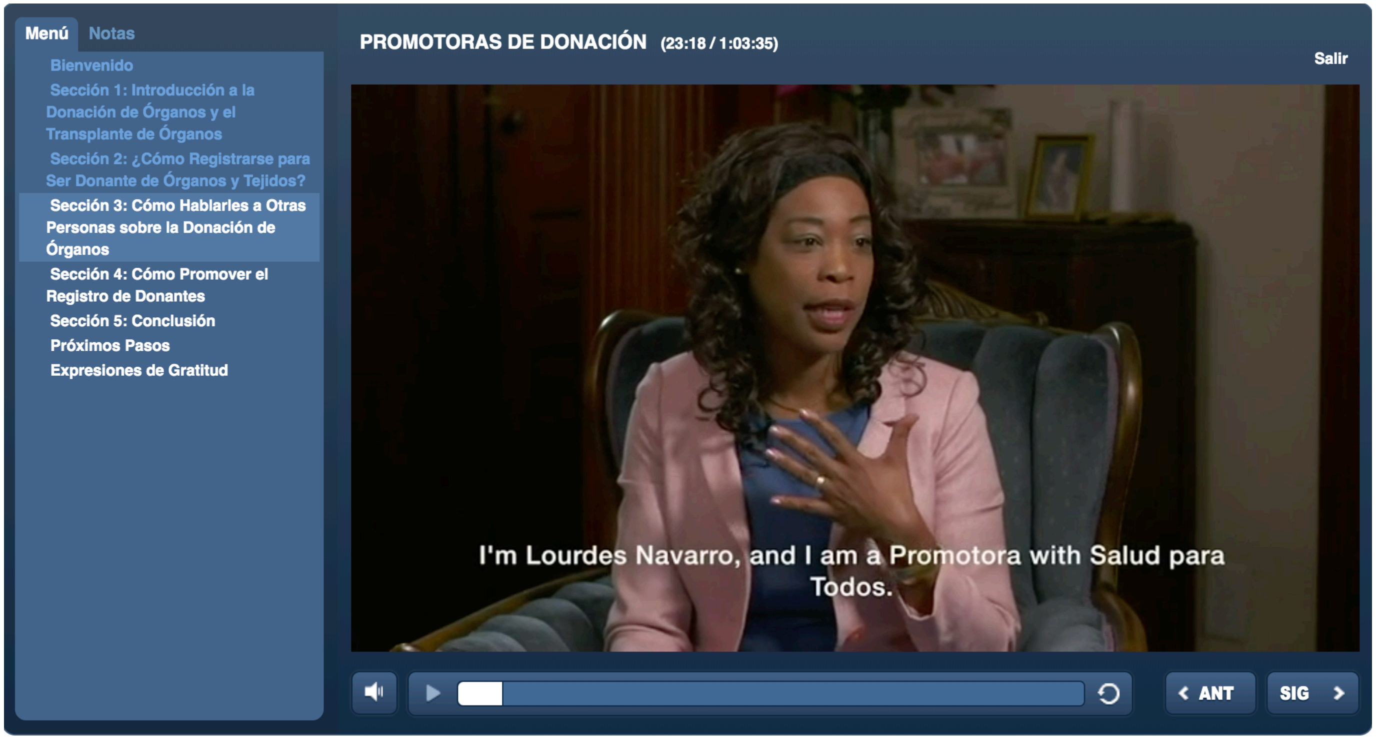

Screenshot 8

The interview with a liver transplant recipient

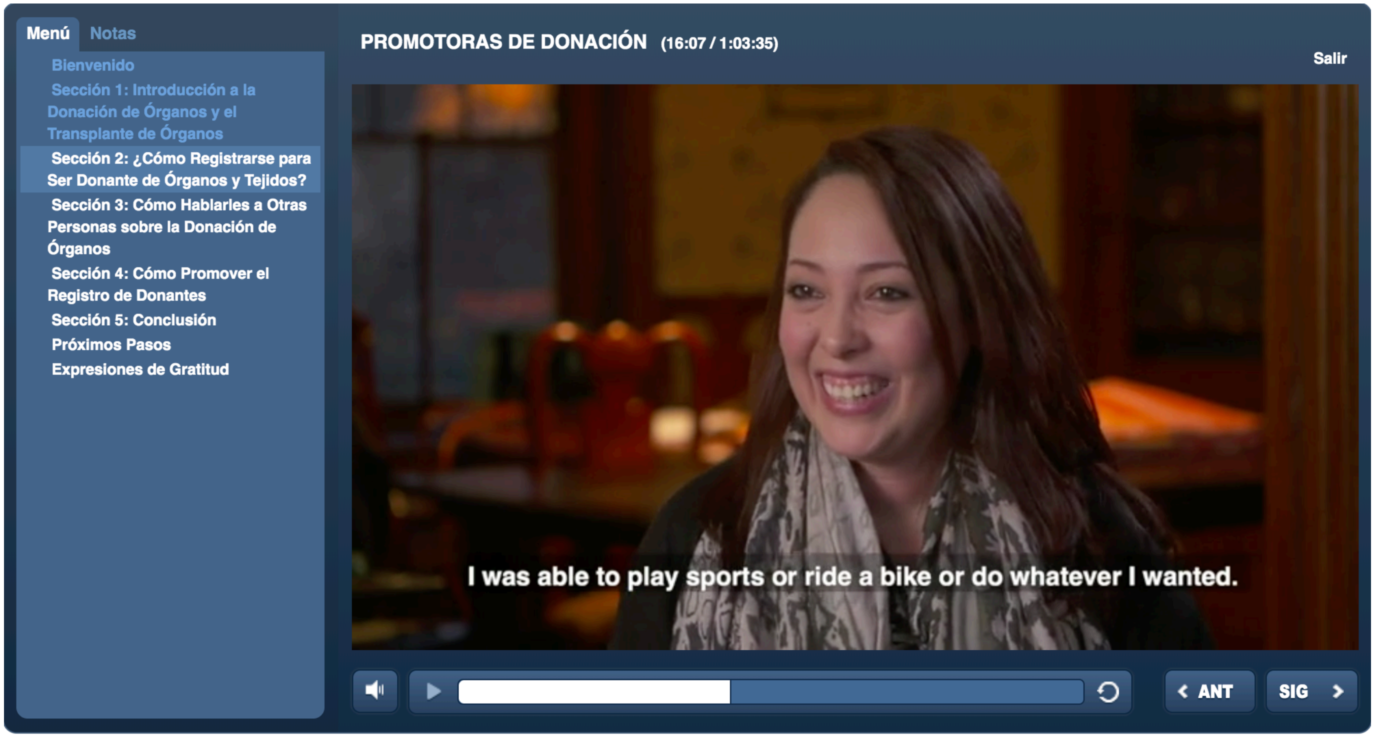

Screenshot 9

The interview with a kidney transplant expert

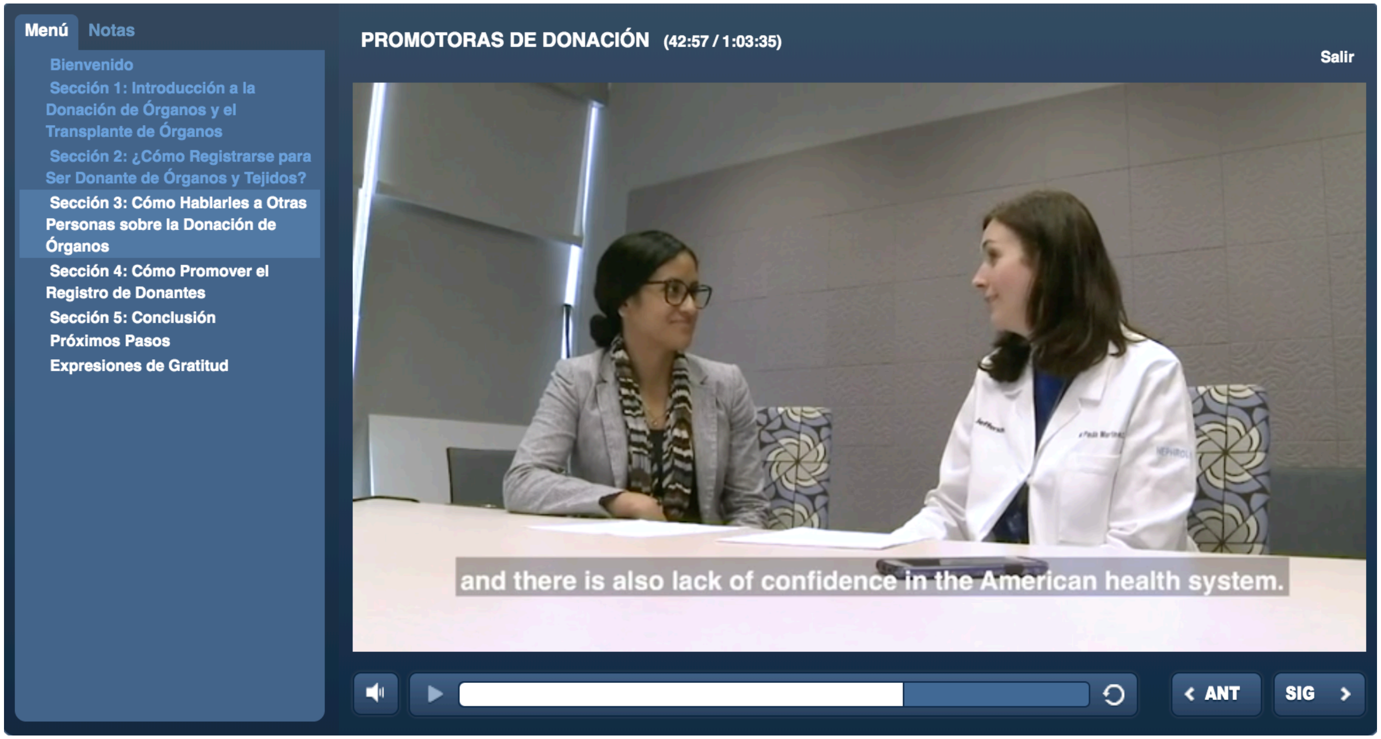

Screenshot 10

The interview with the mother of a deceased organ donor

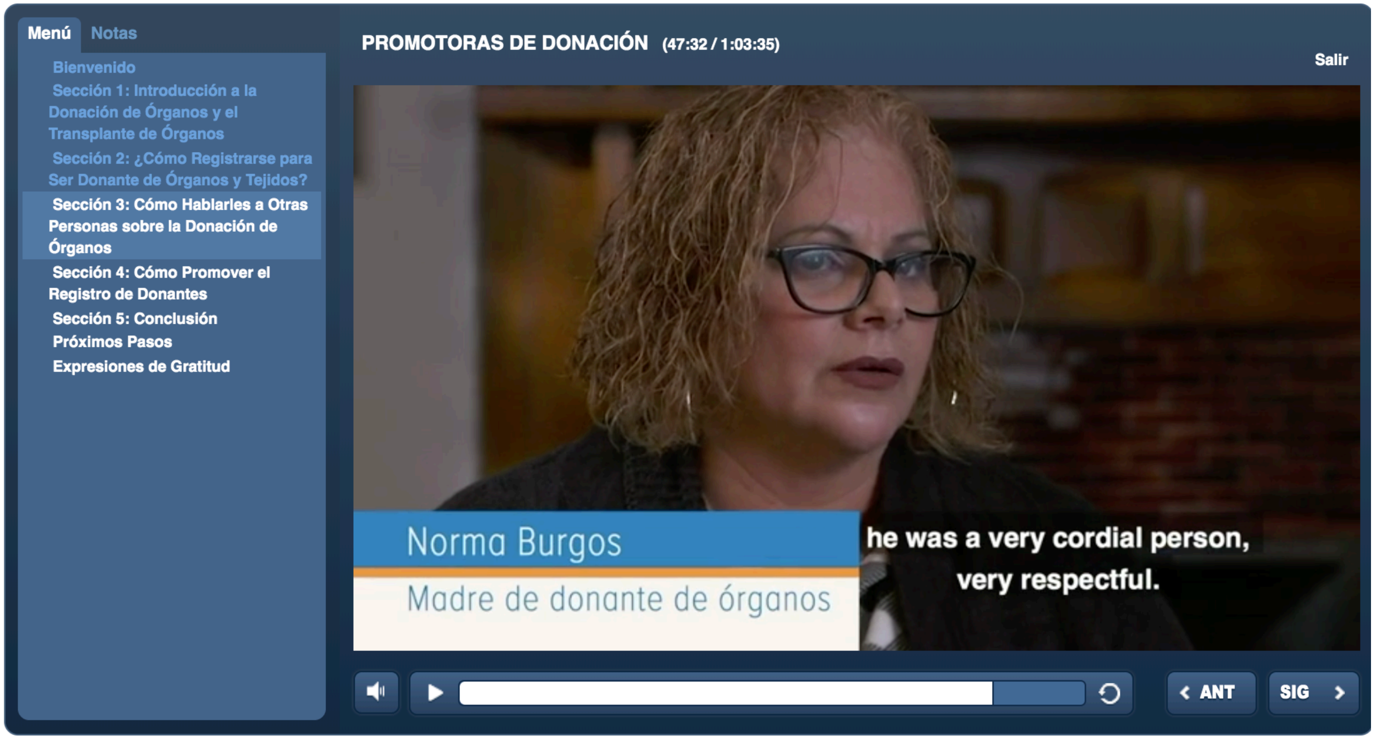

Screenshot 11

The narrator overviewing the key skills necessary to facilitate a *platica* about organ donation and registration

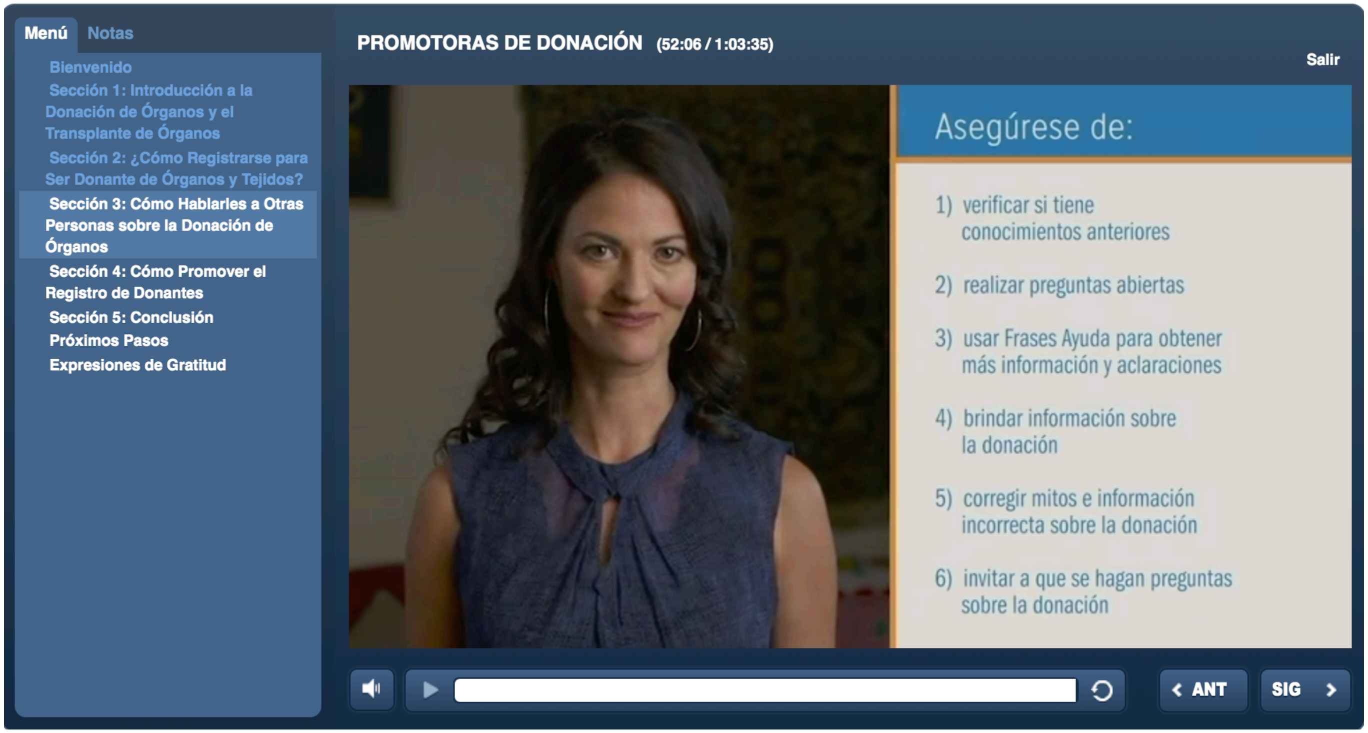

Screenshot 12

The *promotora* demonstrating a key skill during the *platica* dramatization with a sidebar that reiterates the key skill

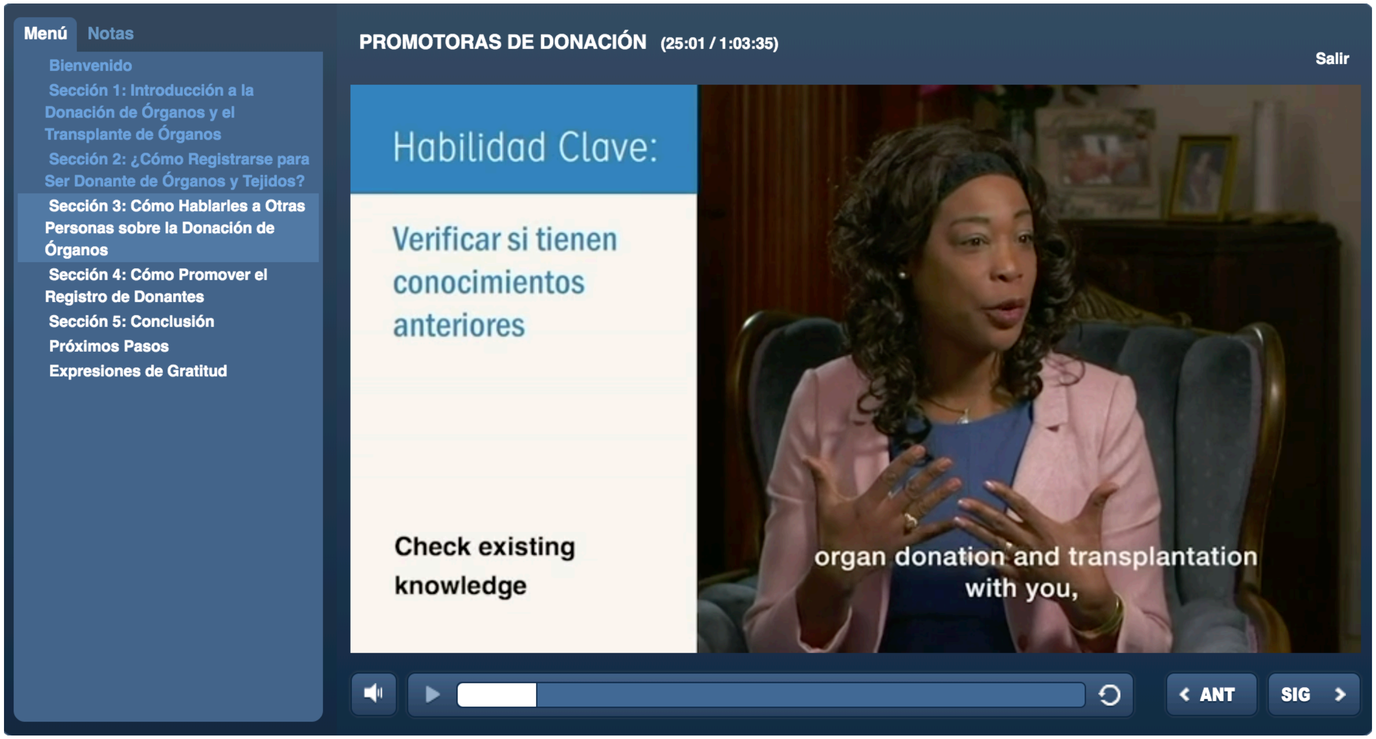

Screenshot 13

Overview of key skills to promote organ donation and registration

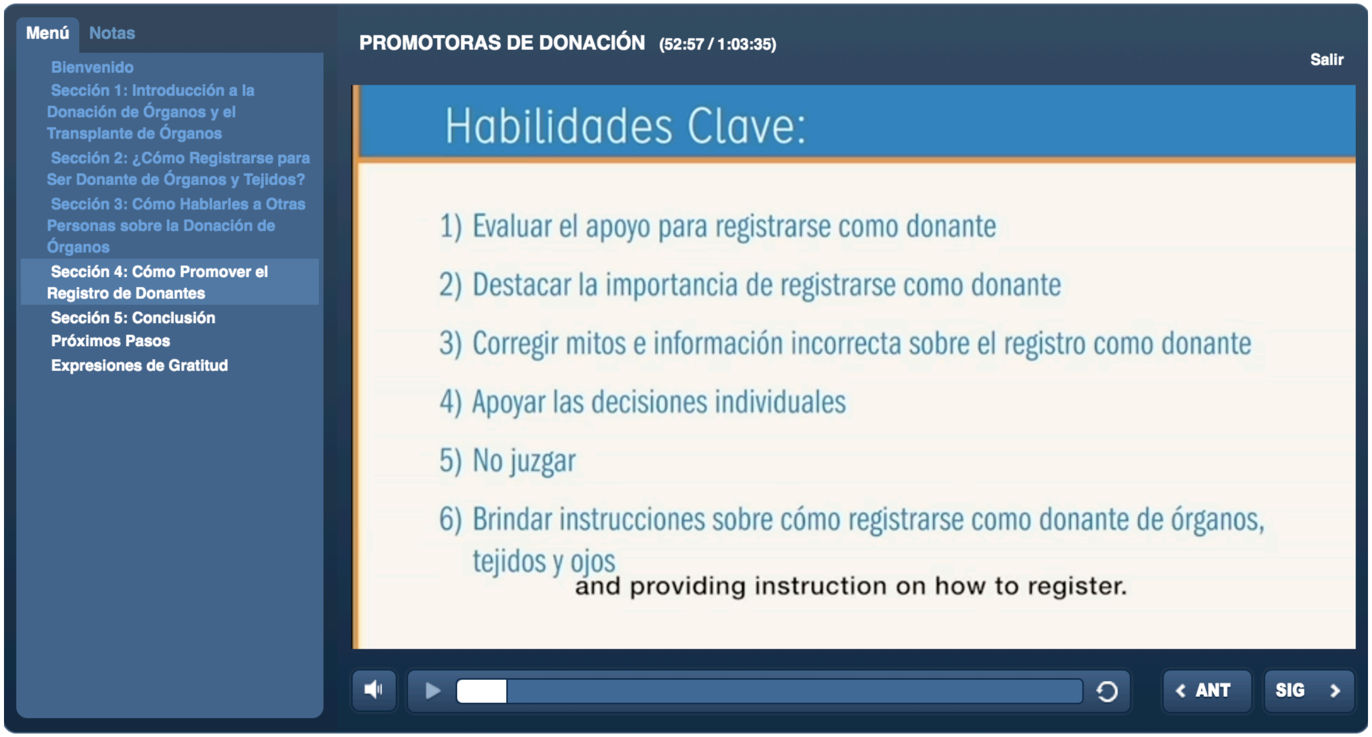

Screenshot 14

Example true/false supplemental activity question

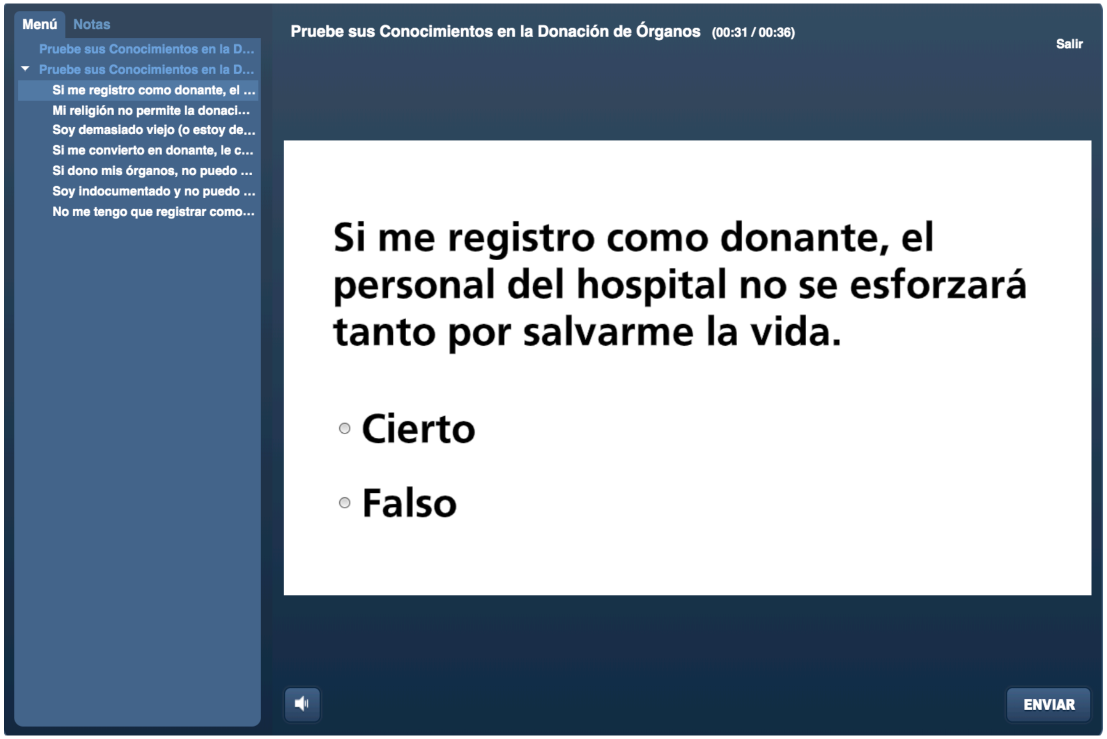

Screenshot 15

Example true/false supplemental activity 'correct' response affirmation

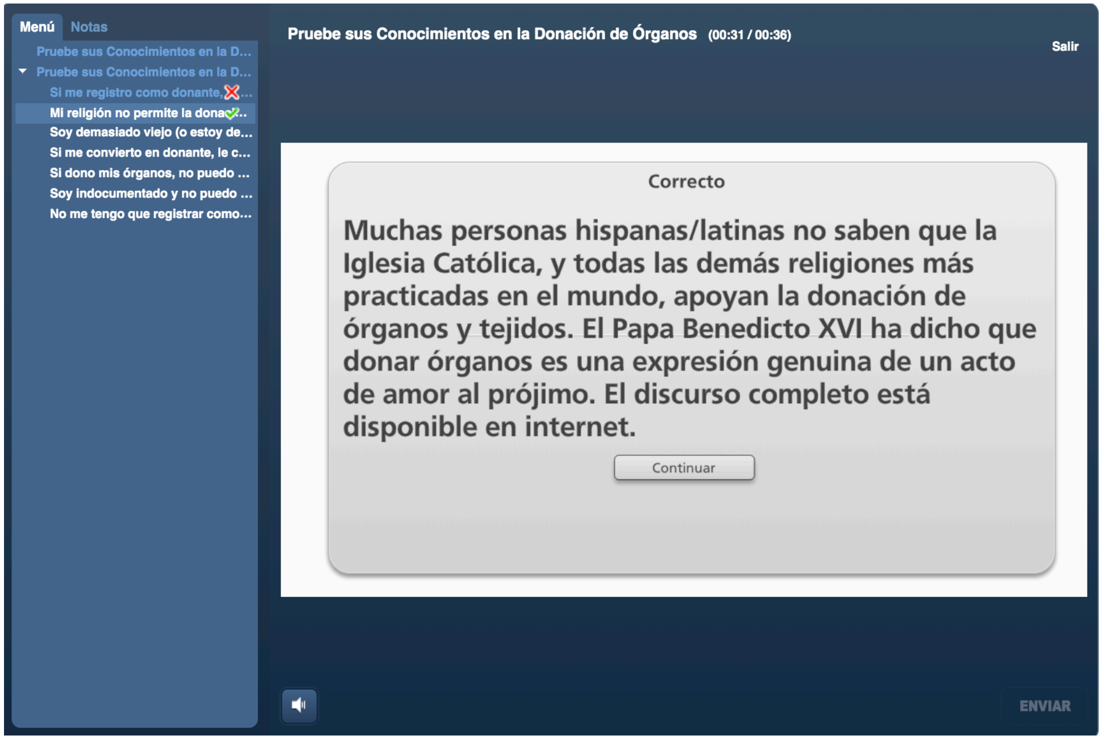

Screenshot 16

Example true/false supplemental activity ‘incorrect’ response explanation

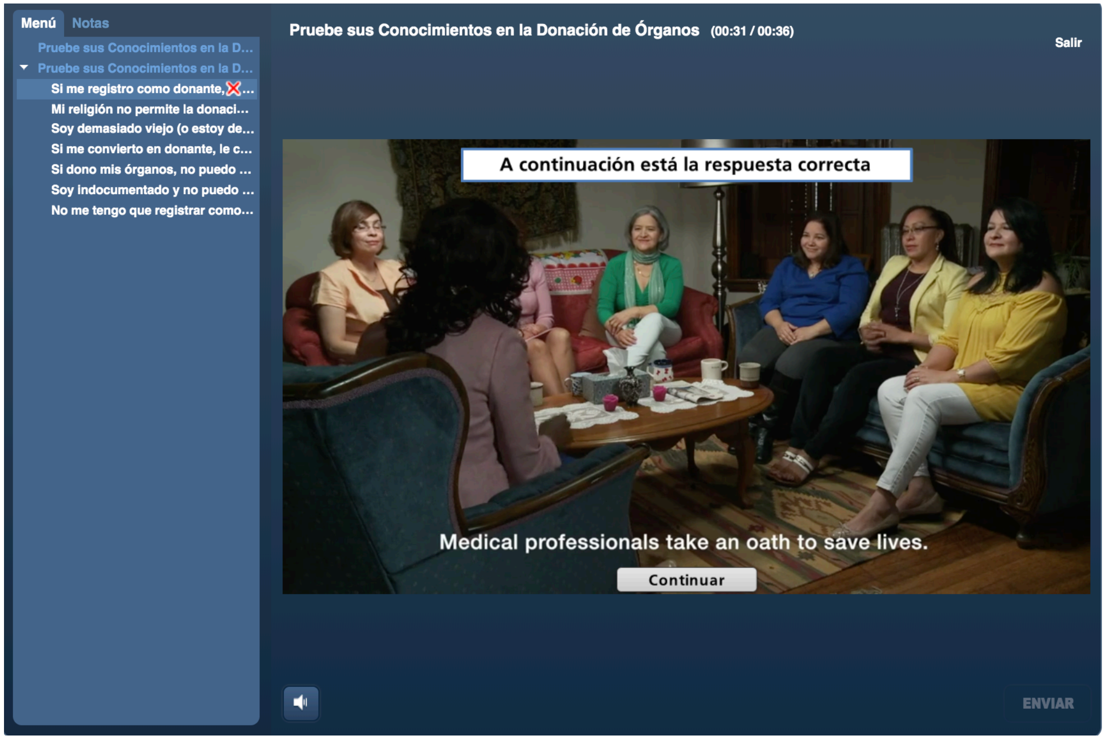

Screenshot 17

Example certificate of completion

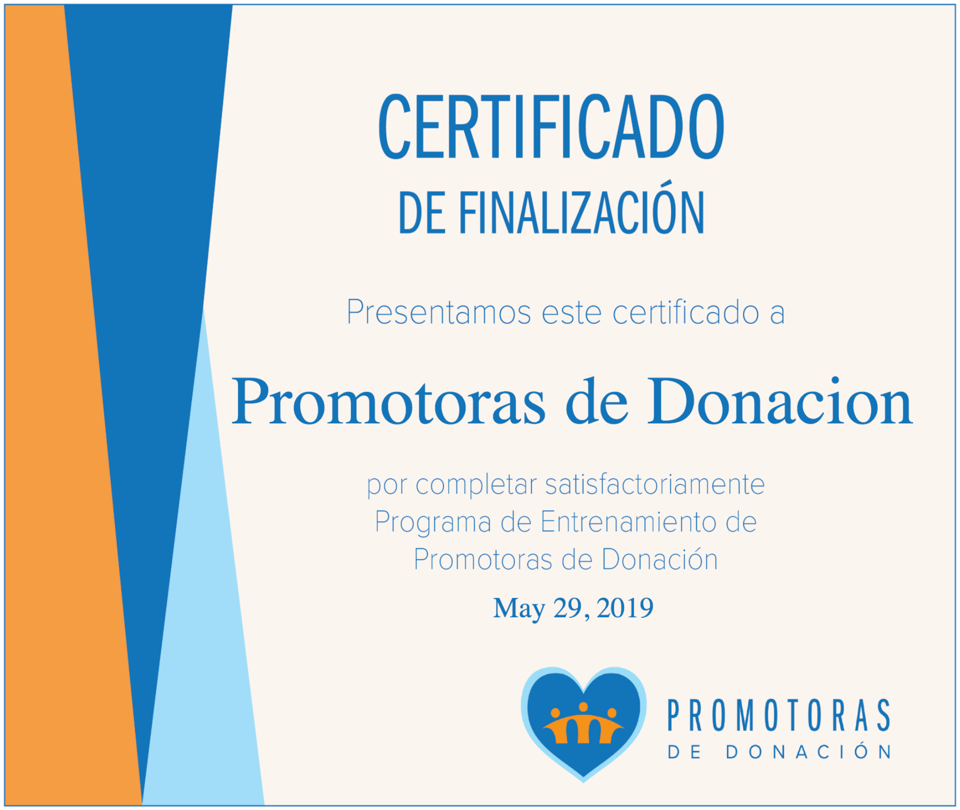
CERTIFICADO

DE FINALIZACIÓN

Presentamos este certificado a

Promotoras de Donacion

por completar satisfactoriamente
Programa de Entrenamiento de
Promotoras de Donación

May 29, 2019

PROMOTORAS
DE DONACIÓN

Supplemental Video 1

How to register as an organ and tissue donor

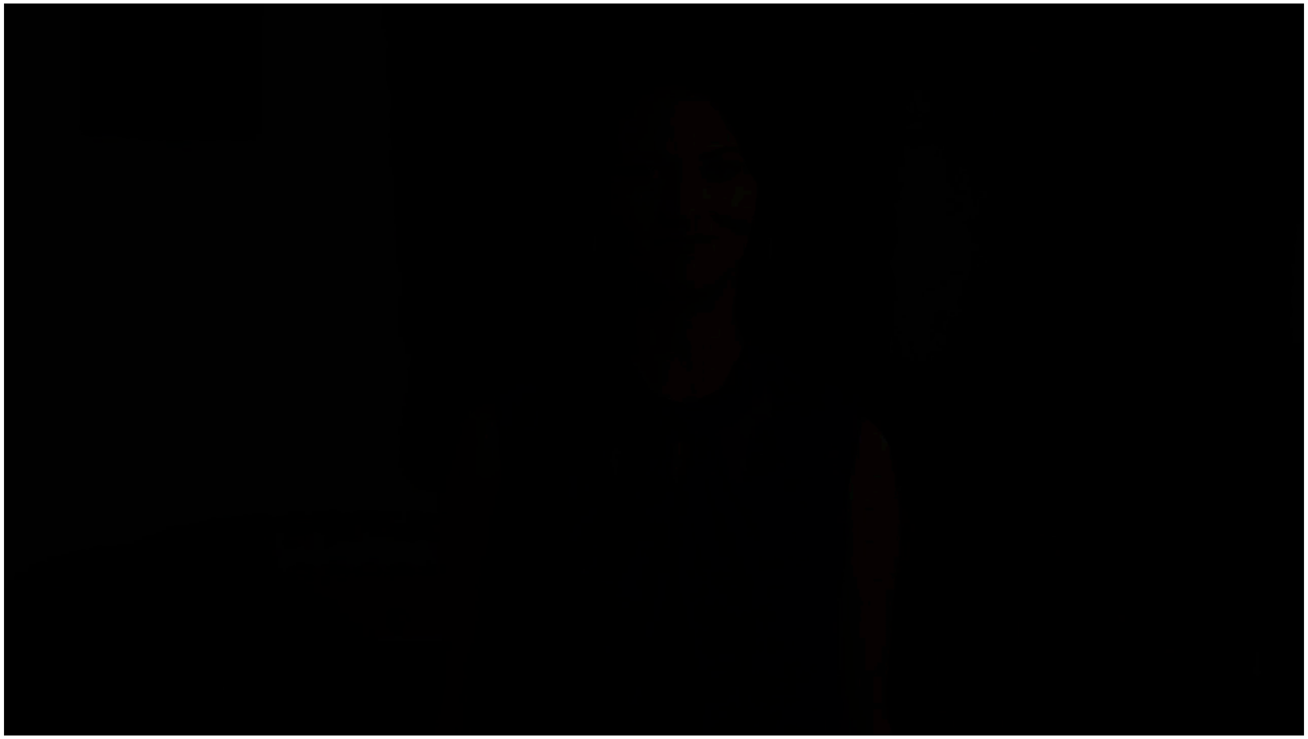

Supplement: Multimedia Appendix 1 [file jmir_v22i1e15793_app1.pdf]
