# Supplementary material for: A Culturally Targeted eLearning Module on Organ Donation (Promotoras de Donación): Design and Development
Source: J Med Internet Res. 2020 Jan 13;22(1):e15793. doi: 10.2196/15793 (PMC6996759; doi:10.2196/15793)
Supplement: Multimedia Appendix 2 [file jmir_v22i1e15793_app2.pdf]

Multimedia Appendix 2: Beta Test Survey – *Promotoras De Donaión* e-Learning Module

Thank you for completing the Promotoras de Donación e-Learning Module! Your feedback is important for helping us improve the quality of the module. Please take a moment to complete this short survey about the module.

These first questions ask you to rate aspects of the module you just completed using a scale that ranges from strongly disagree to strongly agree.

		**Strongly Disagree**				**Strongly Agree**
1a.	The module presented information that was new to me.	ρ	ρ	ρ	ρ	ρ
b.	The module was too long.	ρ	ρ	ρ	ρ	ρ
c.	I enjoyed the activities in the module.	ρ	ρ	ρ	ρ	ρ
d.	The activities in the module helped me to learn.	ρ	ρ	ρ	ρ	ρ
e.	The module was well-organized.	ρ	ρ	ρ	ρ	ρ
f.	I will use what I learned in this module to talk with my community about organ transplantation and donor registration.	ρ	ρ	ρ	ρ	ρ
g.	The module covered too much material.	ρ	ρ	ρ	ρ	ρ
h.	The conversation about organ donation seemed realistic.	ρ	ρ	ρ	ρ	ρ
i.	It was helpful to see an example of a conversation about organ donation.	ρ	ρ	ρ	ρ	ρ
j.	I found the material covered in the module interesting.	ρ	ρ	ρ	ρ	ρ
k.	After completing the module, I feel ready to teach others about organ donation and transplantation.	ρ	ρ	ρ	ρ	ρ
l.	I would recommend this training to other *Promotoras*.	ρ	ρ	ρ	ρ	ρ
m.	The module took a reasonable amount of time to finish.	ρ	ρ	ρ	ρ	ρ

The next questions ask about more evaluation questions about the module, including how well the module prepares you to serve your community using a scale that ranges from strongly disagree to strongly agree.

		**Strongly Disagree**	**Disagree**		**Strongly Agree**
2a.	The words, phrases, and expressions used in the module are too technical (too much jargon) for the average Promotora.	ρ	ρ	ρ	ρ
b.	The words, phrases, and expressions used in module were free from stereotypes about the Hispanic/Latina community.	ρ	ρ	ρ	ρ
c.	People in my community would believe that the messages in the module are from credible sources.	ρ	ρ	ρ	ρ
d.	The messages in the module address organ donation and transplantation myths that are common in my community.	ρ	ρ	ρ	ρ
e.	The people in the module physically look like people in my community (hair style, clothes, etc.).	ρ	ρ	ρ	ρ

These next questions ask about your ability to easily use the website and module using a scale that ranges from strongly disagree to strongly agree.

		**Strongly Disagree**	**Disagree**	**Agree**	**Strongly Agree**
3a.	The website was too complex.	ρ	ρ	ρ	ρ
b.	The website was easy to use.	ρ	ρ	ρ	ρ
c.	I liked the look and feel of the website.	ρ	ρ	ρ	ρ
d.	It was easy to save my place in the module and finish the module later.	ρ	ρ	ρ	ρ
e.	I would imagine that most *Promotoras* would find the website easy to use.	ρ	ρ	ρ	ρ

4. We had originally intended for the module to take about 40 minutes to complete. If we were to remove sections of the video to shorten its length, which would you recommend cutting:

	Definitely Remove	Maybe Remove	Definitely do not Remove
a. Video of man and dog	ρ	ρ	ρ
b. Animation describing three ways to donate	ρ	ρ	ρ
c. Testimonial from Carmen Rosa (liver transplant recipient)	ρ	ρ	ρ
d. Transplant expert talking about the need for donors	ρ	ρ	ρ
e. Testimonial from Norma Burgos (mother of organ donor)	ρ	ρ	ρ
f. Video of Promotora discussing organ donation with a group of women	ρ	ρ	ρ
g. Video of Promotora discussing donor registration with a group of women	ρ	ρ	ρ
h. True/False quiz	ρ	ρ	ρ

5. Please use the space below to write down any suggestions on ways to improve the training program:

___

___

___

6. What, if anything, was missing or did not feel right for you in this module?

___

___

___

7. What do you think is the most important message in this video for people who are unfamiliar with the topic of organ donation and transplantation

___

___

___

8. What did you like most about the module?

___

____

____

9. What did you like least about the module?

____

____

____

10. Overall, how would you rate the quality of the module?

**Poor**	**Fair**	**Good**	**Very Good**	**Excellent**
ρ	ρ	ρ	ρ	ρ

These last questions are about you.

11. What term best describes your job role?

- ❑ Leadership
- ❑ Administration
- ❑ Community Health Worker (Promotora)
- ❑ Other ____________________

12. How long have you been working in this role?

_______ years _______ months

13su. What language do you use at home?

- ❑ English only
- ❑ Spanish only
- ❑ Mostly English – some Spanish
- ❑ Mostly Spanish – some English
- ❑ Spanish and English equally

Thank you for taking the time to review the module and complete this survey.
